# Supplementary material for: Disgust and Self-Disgust in Eating Disorders: A Systematic Review and Meta-Analysis
Source: Nutrients. 2022 Apr 21;14(9):1728. doi: 10.3390/nu14091728 (PMC9102838; doi:10.3390/nu14091728)
Supplement: Supplementary file 1 [file nutrients-14-01728-s001.zip › nutrients-1677389-supplementary.pdf]

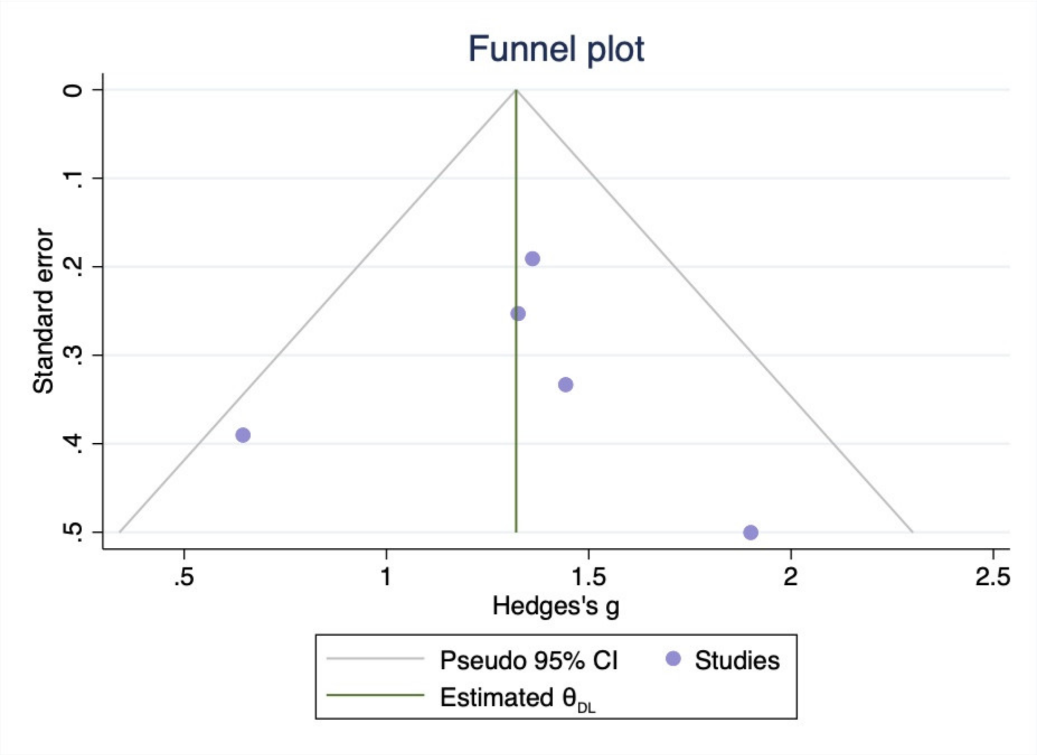

Figure S1. Funnel plot of studies of disgust elicited by food images in EDs versus HCs

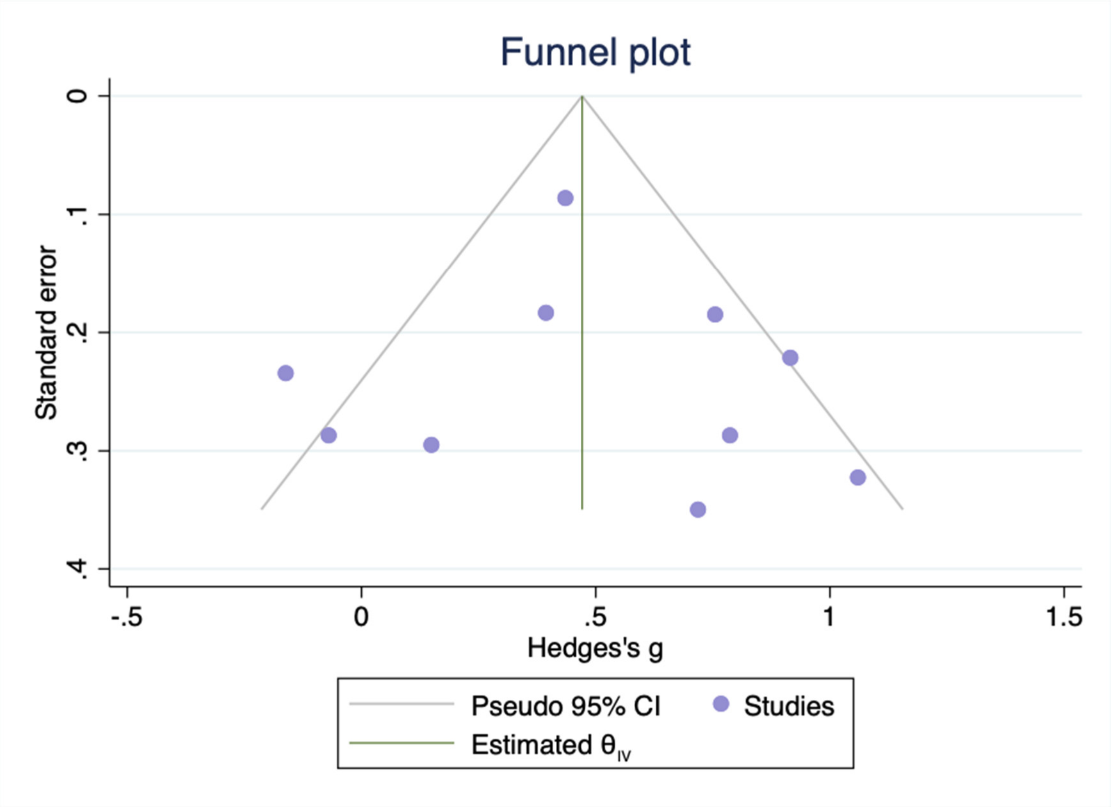

Figure S2. Funnel plot of studies of generic disgust sensitivity in EDs versus HCs

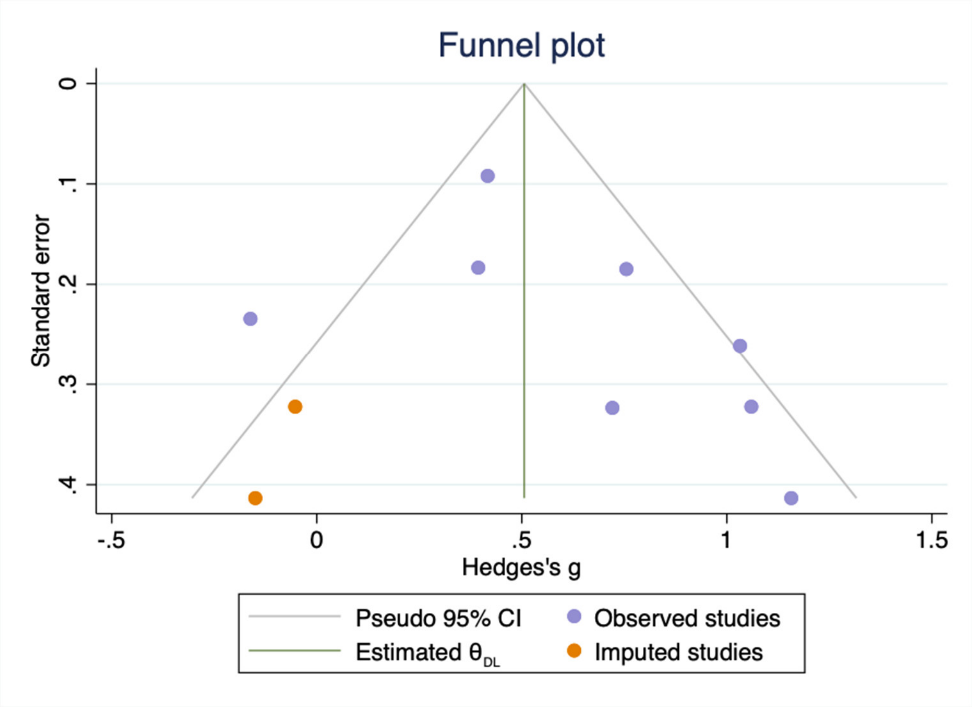

Figure S3. Funnel plot of studies of generic disgust sensitivity in AN versus HCs after applying a trim-and-fill method

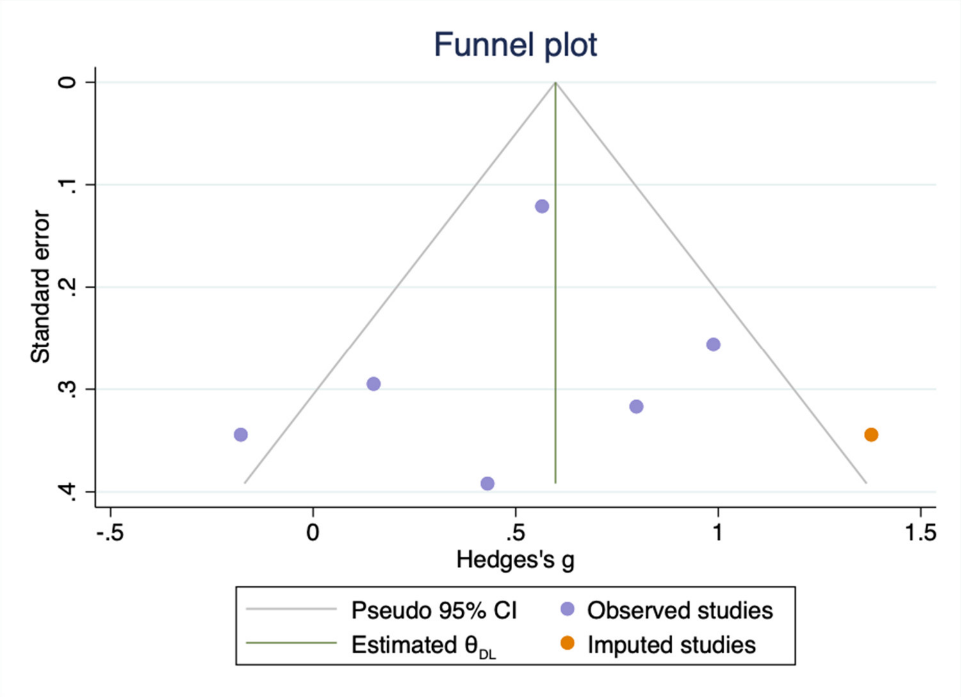

Figure S4. Funnel plot of studies of generic disgust sensitivity in BN and HCs after applying a trim-and-fill method

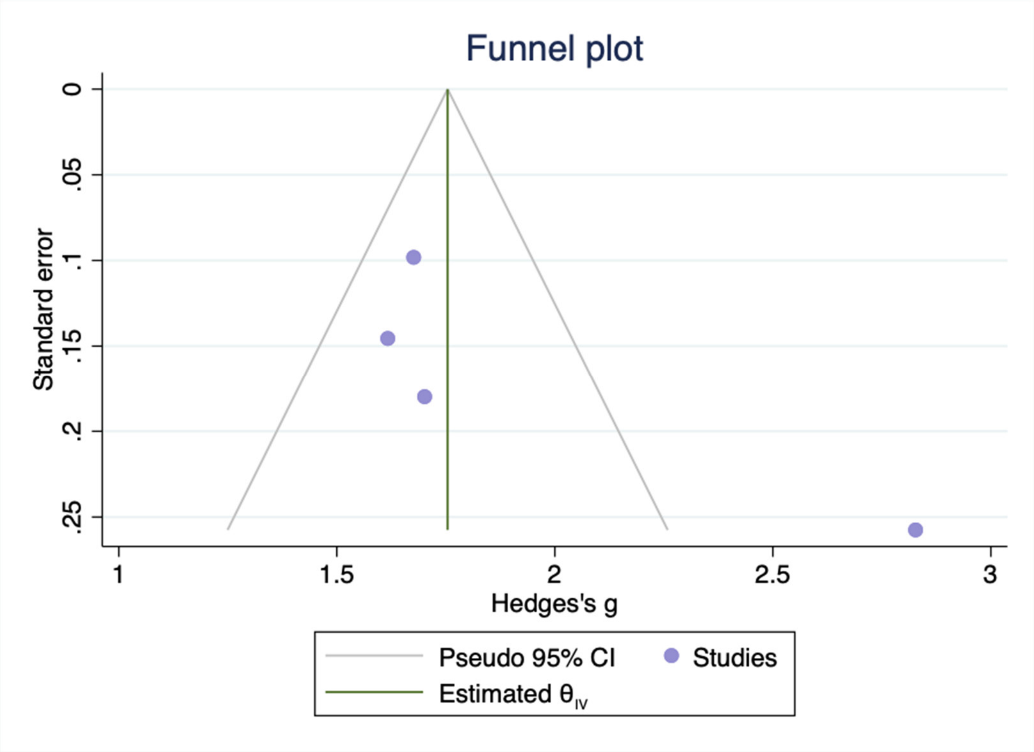

Figure S5. Funnel plot of studies of self-disgust in EDs versus HCs

Table S1. PRISMA (2020) main checklist

**Topic**	**No.**	**Item**	**Location where item is reported**
**TITLE**			
**Title**	1	Identify the report as a systematic review.	1
**ABSTRACT**			
**Abstract**	2	See the PRISMA 2020 for Abstracts checklist	
**INTRODUCTION**			
**Rationale**	3	Describe the rationale for the review in the context of existing knowledge.	1
**Objectives**	4	Provide an explicit statement of the objective(s) or question(s) the review addresses.	1
**METHODS**			

**Eligibility criteria**	5	Specify the inclusion and exclusion criteria for the review and how studies were grouped for the syntheses.	2, 3
**Information sources**	6	Specify all databases, registers, websites, organisations, reference lists and other sources searched or consulted to identify studies. Specify the date when each source was last searched or consulted.	2, 3
**Search strategy**	7	Present the full search strategies for all databases, registers and websites, including any filters and limits used.	Table S3
**Selection process**	8	Specify the methods used to decide whether a study met the inclusion criteria of the review, including how many reviewers screened each record and each report retrieved, whether they worked independently, and if applicable, details of automation tools used in the process.	2, 3, 5
**Data collection process**	9	Specify the methods used to collect data from reports, including how many reviewers collected data from each report, whether they worked independently, any processes for obtaining or confirming data from study investigators, and if applicable, details of automation tools used in the process.	2, 3, 5
**Data items**	10a	List and define all outcomes for which data were sought. Specify whether all results that were compatible with each outcome domain in each study were sought (e.g. for all measures, time points, analyses), and if not, the methods used to decide which results to collect.	2, 3, 5
	10b	List and define all other variables for which data were sought (e.g. participant and intervention characteristics, funding sources). Describe any assumptions made about any missing or unclear information.	2, 3, 5
**Study risk of bias assessment**	11	Specify the methods used to assess risk of bias in the included studies, including details of the tool(s) used, how many reviewers assessed each study and whether they worked independently,	4

		and if applicable, details of automation tools used in the process.	
**Effect measures**	12	Specify for each outcome the effect measure(s) (e.g. risk ratio, mean difference) used in the synthesis or presentation of results.	2, 3, 5
**Synthesis methods**	13a	Describe the processes used to decide which studies were eligible for each synthesis (e.g. tabulating the study intervention characteristics and comparing against the planned groups for each synthesis (item 5)).	2, 3, 5
	13b	Describe any methods required to prepare the data for presentation or synthesis, such as handling of missing summary statistics, or data conversions.	2, 3, 5
	13c	Describe any methods used to tabulate or visually display results of individual studies and syntheses.	2, 3, 5
	13d	Describe any methods used to synthesize results and provide a rationale for the choice(s). If meta-analysis was performed, describe the model(s), method(s) to identify the presence and extent of statistical heterogeneity, and software package(s) used.	2, 3, 5
	13e	Describe any methods used to explore possible causes of heterogeneity among study results (e.g. subgroup analysis, meta-regression).	2, 3, 5
	13f	Describe any sensitivity analyses conducted to assess robustness of the synthesized results.	2, 3, 5
**Reporting bias assessment**	14	Describe any methods used to assess risk of bias due to missing results in a synthesis (arising from reporting biases).	5
**Certainty assessment**	15	Describe any methods used to assess certainty (or confidence) in the body of evidence for an outcome.	5
**RESULTS**			

**Study selection**	16a	Describe the results of the search and selection process, from the number of records identified in the search to the number of studies included in the review, ideally using a flow diagram.	5
	16b	Cite studies that might appear to meet the inclusion criteria, but which were excluded, and explain why they were excluded.	5
**Study characteristics**	17	Cite each included study and present its characteristics.	5
**Risk of bias in studies**	18	Present assessments of risk of bias for each included study.	Tables S4, S5, S6, S7, S8
**Results of individual studies**	19	For all outcomes, present, for each study: (a) summary statistics for each group (where appropriate) and (b) an effect estimate and its precision (e.g. confidence/credible interval), ideally using structured tables or plots.	Tables 1, 2, 3, 4, 5
**Results of syntheses**	20a	For each synthesis, briefly summarise the characteristics and risk of bias among contributing studies.	5, Figures 2, 3, 4, 5, 6
	20b	Present results of all statistical syntheses conducted. If meta-analysis was done, present for each the summary estimate and its precision (e.g. confidence/credible interval) and measures of statistical heterogeneity. If comparing groups, describe the direction of the effect.	5, Figures 2, 3, 4, 5, 6
	20c	Present results of all investigations of possible causes of heterogeneity among study results.	5,
	20d	Present results of all sensitivity analyses conducted to assess the robustness of the synthesized results.	5, Figures S1, S2, S3, S4, S5
**Reporting biases**	21	Present assessments of risk of bias due to missing results (arising from reporting biases) for each synthesis assessed.	5, Figures S1, S2, S3, S4, S5
**Certainty of evidence**	22	Present assessments of certainty (or confidence) in the body of evidence for each outcome assessed.	5, Figures S1, S2, S3, S4, S5

**DISCUSSION**			
**Discussion**	23a	Provide a general interpretation of the results in the context of other evidence.	6
	23b	Discuss any limitations of the evidence included in the review.	6
	23c	Discuss any limitations of the review processes used.	6
	23d	Discuss implications of the results for practice, policy, and future research.	6
**OTHER INFORMATION**			
**Registration and protocol**	24a	Provide registration information for the review, including register name and registration number, or state that the review was not registered.	2
	24b	Indicate where the review protocol can be accessed, or state that a protocol was not prepared.	2
	24c	Describe and explain any amendments to information provided at registration or in the protocol.	2
**Support**	25	Describe sources of financial or non-financial support for the review, and the role of the funders or sponsors in the review.	Page 33
**Competing interests**	26	Declare any competing interests of review authors.	Page 33
**Availability of data, code and other materials**	27	Report which of the following are publicly available and where they can be found: template data collection forms; data extracted from included studies; data used for all analyses; analytic code; any other materials used in the review.	Page 33

Table S2. PRISMA (2020) abstract checklist

Title			
**Title**	1	Identify the report as a systematic review.	Yes
**Background**			
**Objectives**	2	Provide an explicit statement of the main objective(s) or question(s) the review addresses.	Yes
**Methods**			
**Eligibility Criteria**	3	Specify the inclusion and exclusion criteria for the review.	Yes
**Information Sources**	4	Specify the information sources (e.g. databases, registers) used to identify studies and the date when each was last searched.	Yes
**Risk Of Bias**	5	Specify the methods used to assess risk of bias in the included studies.	Yes
**Synthesis Of Results**	6	Specify the methods used to present and synthesize results.	Yes
**Results**			
**Included Studies**	7	Give the total number of included studies and participants and summarise relevant characteristics of studies.	Yes
**Synthesis Of Results**	8	Present results for main outcomes, preferably indicating the number of included studies and participants for each. If meta-analysis was done, report the summary estimate and confidence/credible interval. If comparing groups, indicate the direction of the effect (i.e. which group is favoured).	Yes
**Discussion**			
**Limitations Of Evidence**	9	Provide a brief summary of the limitations of the evidence included in the review (e.g. study risk of bias, inconsistency and imprecision).	Yes
**Interpretation**	10	Provide a general interpretation of the results and important implications.	Yes
**Other**			
**Funding**	11	Specify the primary source of funding for the review.	Yes

**Registration**	12	Provide the register name and registration number.	Yes

Table S3. Search terms used in the systematic search of the electronic databases.

Databases and Search Strategies	Articles
**PubMed**<br>(Disgust OR “self-disgust”) AND (“eating disorders” OR “anorexia nervosa” OR “anorexia” OR “bulimia nervosa” OR bulimia OR “binge eating” OR “binge eating disorder” OR ARFID OR “avoidant restrictive food intake disorder” OR “avoidant/restrictive food intake disorder” OR pica OR rumination)	130
**Web of Science**<br>TS= (Disgust OR “self-disgust”) AND TS= (“eating disorders” OR “anorexia nervosa” OR “anorexia” OR “bulimia nervosa” OR bulimia OR “binge eating” OR “binge eating disorder” OR ARFID OR “avoidant restrictive food intake disorder” OR “avoidant/restrictive food intake disorder” OR pica OR rumination)	161
**APA PsycInfo (Ovid)**<br>((disgust or self-disgust) and (eating disorders or anorexia nervosa or anorexia or bulimia nervosa or bulimia or binge eating or binge eating disorder or ARFID or avoidant restrictive food intake disorder or avoidant restrictive food intake disorder or pica or rumination)).mp	233

Table S4. Quality assessment of cross-sectional studies (N=4)

	Were the criteria for inclusion in the sample clearly defined?	Were the study subjects and the setting described in detail?	Was the exposure measured in a valid and reliable way?	Were objective, standard criteria used for measurement of the condition?	Were confounding factors identified?	Were strategies to deal with confounding factors stated?	Were the outcomes measured in a valid and reliable way?	Was appropriate statistical analysis used?	Overall Appraisal	Comment
**Cooper et al. (1988) [41]**	Y	Y	N.A.	Y	N	N	Y	Y	Include	
**Fox & Froom (2009) [54]**	Y	Y	N.A.	Y	Y	Y	Y	Y	Include	
**Hildebrandt et al. (2018) [73]**	Y	Y	Y	U	N	N	Y	Y	Include	
**Richson et al. (2020) [42]**	Y	Y	N.A.	Y	N	N	Y	Y	Include	

Abbreviations: Y: Yes, N: No, U: Unclear, N/A: Not Applicable.
Footnotes: * Disgust was a part of experiment or experimental task.

Table S5. Quality assessment of case-control studies (N=42)

	**Were the groups comparable other than the presence of disease in cases or the absence of disease in controls?**	**Were cases and controls matched appropriately?**	**Were the same criteria used for identification of cases and controls?**	**Was exposure measured in a standard, valid and reliable way?**	**Was exposure measured in the same way for cases and controls?**	**Were confounding factors identified?**	**Were strategies to deal with confounding factors stated?**	**Were outcomes assessed in a standard, valid and reliable way for cases and controls?**	**Was the exposure period of interest long enough to be meaningful?**	**Was appropriate statistical analysis used?**	**Overall Appraisal**	**Comment**
**Aharoni & Hertz (2012) [35]**	Y	Y	Y	N.A.	N.A.	Y	Y	Y	N.A.	Y	Include	
**Ashworth et al. (2011) [78]**	Y	Y	Y	Y	Y	Y	Y	*	Y	Y	Include	

**Bell et al. (2017)** [**33**]	U	N	Y	N.A.	N.A.	Y	Y	Y	N.A.	Y	Include
**Bornholt et al. (2005)** [**28**]	Y	U	Y	N.A.	N.A.	Y	Y	Y	N.A.	Y	Include
**Buvat-Herbaut et al. (1983)** [**29**]	Y	U	Y	N.A.	N.A.	N	N	Y	N.A.	Y	Include
**Davey et al. (1998)** [**39**]	Y	U	Y	N.A.	N.A.	N	N	Y	N.A.	Y	Include
**Dapelo et al. (2016a)** [**75**]	Y	Y	Y	Y	Y	Y	Y	*	Y	Y	Include
**Dapelo et al. (2016b)** [**68**]	Y	Y	Y	Y	Y	N	N	*	Y	Y	Include
**Dapelo et al. (2017)** [**69**]	Y	U	Y	Y	Y	N	N	Y	Y	Y	Include

**Duriez et al. (2021) [67]**	Y	U	Y	Y	Y	N	N	*	Y	Y	Include	
**Fox et al. (2013) [36]**	Y	Y	Y	Y	Y	Y	Y	Y	Y	Y	Include	
**Foroughi et al. (2020) [45]**	Y	Y	Y	Y	Y	Y	Y	Y	Y	Y	Include	Case control + Longitudinal study
**Fujiwara et al. (2017) [74]**	Y	Y	Y	Y	Y	Y	Y	*	Y	Y	Include	
**Gagnon et al. (2018) [56]**	Y	U	Y	Y	Y	Y	Y	*	Y	Y	Include	
**Hay & Katsikitis (2014) [46]**	Y	U	Y	Y	Y	Y	Y	Y	Y	Y	Include	
**Hildebrandt et al. (2015) [78]**	Y	U	Y	Y	Y	Y	Y	Y	Y	Y	Include	

**Hildebrandt et al. (2016) [72]**	Y	U	Y	Y	Y	Y	Y	*	Y	Y	Include
**Horndasch et al. (2012) [47]**	Y	U	Y	Y	Y	Y	Y	Y	Y	Y	Include
**Ille et al. (2014) [44]**	Y	Y	Y	N.A.	N.A.	Y	Y	Y	N.A.	Y	Include
**Jänsch et al. (2009) [70]**	Y	Y	Y	Y	Y	Y	Y	*	Y	Y	Include
**Jiang et al. (2019) [32]**	Y	Y	Y	Y	Y	N	N	Y	Y	Y	Include
**Joos et al. (2009) [48]**	Y	Y	Y	Y	Y	Y	Y	Y	Y	Y	Include
**Joos et al. (2011a) [49]**	Y	Y	Y	Y	Y	Y	Y	Y	Y	Y	Include
**Joos et al. (2011b) [50]**	Y	Y	Y	Y	Y	Y	Y	Y	Y	Y	Include

**Joos et al. (2012) [51]**	Y	U	Y	Y	Y	Y	Y	Y	Y	Y	Include
**Kockler et al. (2017) [55]**	Y	U	Y	Y	Y	N	N	Y	Y	Y	Include
**Kollei et al. (2012) [27]**	Y	U	Y	N.A.	N.A.	Y	Y	Y	N.A.	Y	Include
**Kot et al. (2021) [38]**	Y	Y	Y	N.A.	N.A.	Y	Y	Y	N.A.	Y	Include
**Lule et al. (2014) [65]**	Y	Y	Y	Y	Y	N	N	*	Y	Y	Include
**Marques et al. (2021) [43]**	N	U	Y	N.A.	N.A.	Y	Y	Y	N.A.	Y	Include
**Pollatos et al. (2008) [64]**	Y	Y	Y	Y	Y	Y	Y	Y	Y	Y	Include

**Schienle (2003) [30]**	N	Y	Y	N.A.	N.A.	Y	Y	Y	N.A.	Y	Include
**Schienle et al. (2004) [57]**	Y	U	Y	Y	Y	Y	Y	Y	Y	Y	Include
**Schienle et al. (2009) [76]**	Y	U	Y	Y	Y	Y	Y	*	Y	Y	Include
**Soussignan et al. (2010) [79]**	Y	U	Y	Y	Y	Y	Y	Y	Y	Y	Include
**Wyssen et al. (2019) [66]**	Y	Y	Y	Y	Y	Y	Y	*	Y	Y	Include
**Marzola et al. (2020) [37]**	Y	U	Y	Y	Y	Y	N	Y	Y	Y	Include
**Troop et al. (2000) [34]**	N	U	Y	N.A.	N.A.	Y	Y	Y	N.A.	Y	Include

**Troop et al. (2002)** [**40**]	Y	U	Y	N.A.	N.A.	N	N	Y	N.A.	Y	Include
**Uher et al. (2004)** [**52**]	Y	Y	Y	Y	Y	Y	Y	Y	Y	Y	Include
**Uher et al. (2005)** [**53**]	Y	U	Y	Y	Y	N	N	Y	Y	Y	Include
**Zeeck et al. (2011)** [**26**]	Y	Y	Y	N.A.	N.A.	Y	Y	Y	N.A.	Y	Include

Abbreviations: Y: Yes, N: No, U: Unclear, N/A: Not Applicable.
Footnotes: * Disgust was a part of experiment or experimental task

Table S6. Qualitative assessment of qualitative studies (N=5)

	Is there congruity between the stated philosophical perspective and the research methodology?	Is there congruity between the research methodology and the research question or objectives?	Is there congruity between the research methodology and the methods used to collect data?	Is there congruity between the research methodology and the representation and analysis of data?	Is there congruity between the research methodology and the interpretation of results?	Is there a statement locating the researcher culturally or theoretically?	Is the influence of the researcher on the research, and vice-versa, addressed?	Are participants, and their voices, adequately represented?	Is the research ethical according to current criteria or, for recent studies, and is there evidence of ethical approval by an appropriate body?	Do the conclusions drawn in the research report flow from the analysis, or interpretation, of the data?	Overall Appraisal	Comment
**Brooks et al. (1998) [81]**	Y	Y	Y	Y	Y	N	N	Y	N	Y	Include	

**Clancy (2021) [25]**	U	U	U	U	U	N	N	U	N	Y	Exclude
**Espeset et al. (2012) [82]**	Y	Y	Y	Y	Y	N	Y	Y	Y	Y	Include
**Fox (2009) [54]**	Y	Y	Y	Y	Y	Y	U	Y	Y	Y	Include
**McNamara et al. (2008) [80]**	Y	Y	Y	Y	Y	N	N	Y	Y	Y	Include

Abbreviations: Y: Yes, N: No, U: Unclear, N/A: Not Applicable.

Table S7. Quality assessment of experimental studies (N=1)

	**Was true randomization used for assignment of participants to treatment groups?**	**Is it clear in the study what is the "cause" and what is the "effect" (i.e., there is no confusion about which variable comes first)?**	**Were the participants included in any comparisons similar?**	**Were the participants included in any comparisons receiving similar treatment/ care, other than the exposure or intervention of interest?**	**Was there a control group?**	**Were there multiple measurements of the outcome both pre and post the intervention/exposure?**	**Was follow up complete and if not, were differences between groups in terms of their follow up adequately described and analysed?**	**Were the outcomes of participants included in any comparisons measured in the same way?**	**Were outcomes measured in a reliable way?**	**Was appropriate statistical analysis used?**	**Overall Appraisal**	**Comment**
**Schienle et al. (2017) [31]**	Y	Y	Y	U	Y	Y	N/A	Y	Y	Y	Include	

Abbreviations: Y: Yes, N: No, U: Unclear, N/A: Not Applicable.

Table S8. Quality assessment of randomized controled trials (N=1)

	Was true randomization used for assignment of participants to treatment groups?	Was allocation to treatment groups concealed?	Were treatment groups similar at the baseline?	Were participants blind to treatment assignment?	Were those delivering treatment blind to treatment assignment?	Were outcomes assessors blind to treatment assignment?	Were treatment groups treated identically other than the intervention of interest?	Was follow up complete and if not, were differences between groups in terms of their follow up adequately described and analysed?	Were participants analysed in the groups to which they were randomized?	Were outcomes measured in the same way for treatment groups?	Were outcomes measured in a reliable way?	Was appropriate statistical analysis used?	Was the trial design appropriate, and any deviations from the standard RCT design (individual randomization, parallel groups) accounted for in the conduct and analysis of the trial?	Overall Appraisal	Comment
**Kim et al. (2014)** [**71**]	Y	Y	Y	Y	Y	U	Y	N.A.	N.A.	*	Y	Y	Y	Include	

Abbreviations: Y: Yes, N: No, U: Unclear, N/A: Not Applicable.
Footnotes: * Disgust was a part of experiment or experimental task
